# Improving the ecological relevance of aquatic bacterial communities in biodegradability screening assessments

**DOI:** 10.1016/j.scitotenv.2018.01.264

**Published:** 2018-06-15

**Authors:** Timothy J. Martin, Andrew K. Goodhead, Jason R. Snape, Russell J. Davenport

**Affiliations:** a School of Engineering, Cassie Building, Newcastle University, Newcastle upon Tyne NE1 7RU, United Kingdom; b AstraZeneca Global Environment, Mereside, Alderley Park, Macclesfield, Cheshire SK10 4TG, United Kingdom; c School of Life Sciences, Gibbet Hill Campus, The University of Warwick, Coventry, CV4 7AL, United Kingdom

**Keywords:** Exposure assessment, Persistence, PBT, Biodegradation, Environmental risk assessment

## Abstract

Concentrating cells from aqueous samples is a common requirement for the enumeration of biomass, investigations of microbial diversity and detection of relatively rare organisms in the environment. Accurately representing the initial sampled environments in the concentrated cells is of particular importance when the subsequent analyses have tangible environmental, economic and societal consequences, as is the case with environmental exposure and risk assessment of chemicals. This study investigated the potential use of four different cell concentration methods: centrifugation, membrane filtration, tangential flow filtration and column colonisation. These methods were assessed against a series of scientific and practical criteria, including: similarity of concentrated community to initial environmental sample; cell concentration achieved; biodegradation test outcome; sample throughput; and capital and maintenance costs. All methods increased cell concentration by as little as 10-fold to as much as 1000-fold. DGGE and 454 pyrosequencing analysis showed concentrated communities to have >60% similarity to each other, and the initial sample. There was a general trend for a more reliable assessment of 4-nitrophenol biodegradation in 96-well plate biodegradation assays, with increasing cell concentration. Based on the selection criteria, it is recommended that there is not one concentration method fit for all purposes, rather, the appropriate method should be selected on a case-by-case basis. Membrane filtration would be the most suitable method for low sample volumes; the increased throughput capacity of tangential flow filtration renders it most suitable for large volumes; and centrifugation is most suitable for samples with high initial biomass concentrations. The poor similarity in microbial community composition of the column colonised samples compared to the initial samples, suggested a concentration basis; this combined with its low sample throughput precluded this approach for future concentration studies of planktonic bacterial samples.

## Highlights

• Methods suitable for concentrating environmental samples are available;
• Selecting a suitable method is a pragmatic choice, based on the environment sampled;
• Column colonisation appears to introduce bias, possibly promoting biofilm formation;
• Increased inoculum concentrations result in more reliable biodegradation assessment.

## Graphical abstract

**Figure:**
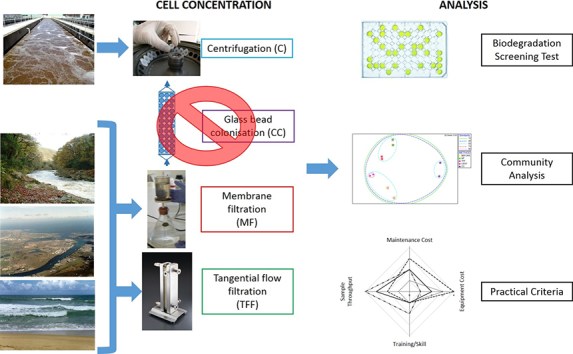

## 1 Introduction

The concentration of cells from aqueous samples is a common prerequisite for the enumeration of bacterioplankton (Hobbie et al., 1977; Glockner et al., 1996; Porter et al., 2004) and biomass (Hobbie et al., 1977), the investigation of microbial diversity and activity (Yannarell and Tripplett, 2004), the cultivation of bacteria (Porter et al., 2004), and the detection of relatively rare bacteria in the environment (Goyal and Gerba, 1980). It is important that the concentrated cells are representative of the initial environment for many of these analyses. It is perhaps most critical when the subsequent analyses have tangible economic, societal, regulatory and environmental consequences, such as the outcome of regulatory biodegradation tests (Boethling et al., 2009; Kowalczyk et al., 2015; Martin et al., 2017a; Martin et al., 2017b). Concentrated bacterial cells have been used as inocula in miniaturized biodegradation tests to investigate the effect of inoculum concentration on bacterial diversity and biodegradation outcome in the context of regulatory chemical risk assessments (Thouand et al., 1995; Goodhead et al., 2013; Martin et al., 2017b). These investigations mimicked international standardized ready biodegradability tests (RBTs) (OECD, 1992) that, for over 30 years, have been the foundation of regulatory frameworks assessing the extent to which chemicals can biodegrade and pose an exposure risk in the environment.

RBTs are used in hazard assessments for classification and labeling, chemical risk assessments, and persistence assessments (ECHA, 2012). A central assumption in all biodegradability tests is that a randomly selected sample from a given environmental compartment will exhibit the requisite microbial diversity (taxonomic and metabolic) representative of that compartment in order to assess the degradability of a compound once it enters the aquatic environment. Standard RBTs (OECD, 1992) are stringent screening tests that use inocula from a range of environmental sources (domestic sewage, activated sludge, secondary wastewater treatment effluent, rivers, sediments, soils and composites) with total cell concentrations ranging over four orders of magnitude (Kowalczyk et al., 2015). In recent years there has been a shift in environmental regulations from identifying those chemicals that can biodegrade in RBTs and assigning measured or default biodegradation half-lives for predicting compartment specific exposure concentrations, to identifying those chemicals that persist in the environment, as these are likely to pose the greatest exposure hazard (Scheringer, 1996). The environmentally unrealistic low bacterial cell concentrations used in RBTs result in highly variable test outcomes (Thouand et al., 1995; Goodhead et al., 2013; Martin et al., 2017b) that frequently report false negative results; i.e. chemicals that are known to biodegrade in the natural environment fail to degrade in these tests because of their high stringency. Indeed false negative results may be responsible for 20–80% of test fails (ECETOC, 2007). A number of scientific workshops have identified the improvement of such biodegradation screening tests as a key priority, especially with respect to persistence assessments (ECETOC, 2003; ECETOC, 2007; ECETOC, 2012; ECETOC, 2017). Solutions that included longer test durations (to encompass persistence half-life thresholds (Boethling et al., 2009)) and the use of environmentally relevant inocula concentrations were advocated based on previous scientific evidence (Nyholm et al., 1984; Nyholm and Kristensen, 1992; Thouand et al., 1995). The enhancement of inocula concentrations to environmentally relevant levels was recommended in REACH guidelines for persistence assessments (EC, 2009; Comber and Holt, 2010), on the basis that it preserves an environmentally realistic microbial diversity that a chemical is likely to encounter in the environment. Indeed, it has recently been shown that the use of environmentally relevant inocula concentrations in biodegradation screening tests, led to an improvement in the reliability of such biodegradation and persistence assessments (Martin et al., 2017b).

In this study, we compared four microbial cell concentration methods of aqueous samples, three of which are commonly used in aquatic biology: membrane filtration (Hobbie et al., 1977; Glockner et al., 1996; Porter et al., 2004; Yannarell and Tripplett, 2004; APHA et al., 2005; Goodhead, 2009), tangential flow filtration (Porter et al., 2004; ECHA, 2012), and centrifugation (OECD, 1992; O'Malley, 2006); and one of which was specifically designed to increase cell concentrations for use in marine biodegradation tests (Mauffret et al., 2009). We assessed each method with respect to the following: the ability to preserve the community structure of the original sample, the magnitude of the increase in cell concentration and the probability of biodegradation of 4-nitrophenol. In addition, we assessed comparative costs, sample throughput and ease of use for each of the concentration methods to determine suitability for regulatory uptake and transferability between research organisations conducting biodegradation assessments.

## 2 Materials and methods

### 2.1 Sample selection

River, estuarine and marine samples were collected from the River Eden at Temple Sowerby, the Tees Estuary at Teesmouth and the English Channel at Plymouth respectively (Fig. S1 SI). Activated sludge samples were not included as their high cell concentration precludes them from filtration techniques. Furthermore, their high specific density (1.02 g mL^−1^) and floc formation naturally lends itself to gravity-based methods such as centrifugation rather than size exclusion as a means of cell concentration. A 10 L environmental sample was collected from each site in sterile plastic carboys. Marine samples were collected using a converted manual bilge pump, from approximately 2–4 m below the surface. River water samples were collected using a sterile collection bucket. Estuary samples were collected by wading into the estuary and sampling as close to the centre of the estuary as feasible using a sterile collection bucket, typically at a depth of 1 m below the surface. Environmental samples were collected in April within 24 h of each other. Samples were kept aerobic at 4 °C and processed within 24 h of collection. Cell concentration was performed at ambient temperature. 5 L of each environmental sample were concentrated to give a nominal 100 × cell concentrate. Total cell counts of all samples, pre- and post-concentration, were determined via epifluorescence microscopy using 4′, 6-diamidino-2-phenylindole (DAPI) (Davenport and Curtis, 2004). Sub-samples of original and concentrated samples were stored at −20 °C for subsequent molecular analysis.

### 2.2 Membrane filtration (MF)

5 L of each sample were concentrated by filtration through sterile 0.22 μm filters (Scientific Laboratory Supplies, Hessle, UK). Filters were replaced when they reached their capacity and were placed in sterile 50 mL centrifuge tubes containing 50 mL filtrate. The filters were then agitated vigorously using a sterile glass rod, to aid the resuspension of biomass in the filtrate. Following settling of the filter remnants, the concentrated sample was then decanted into a new, sterile 50 mL centrifuge tube to produce 50 mL concentrated test inoculum.

### 2.3 Tangential flow filtration (TFF)

5 L environmental sample were pre-filtered through a nylon mesh (Sigma Aldrich, Poole, UK), nominal pore size 10 μm, to remove coarse particles and subsequently processed through a 0.22 μm TFF unit as per manufacturer's instructions (Millipore, Billerica, MA, USA); the permeate was collected and the retentate recycled to the feed tank until the entire sample had been processed as permeate, leaving a suspension of cells on the surface of the filter which were collected by reversing the pump flow direction and backwashing the filter with 50 mL permeate collected in a sterile 50 mL centrifuge tube

### 2.4 Centrifugation (C)

Environmental samples were concentrated by adding 250 mL sample to a sterile centrifuge tube. The sample was centrifuged at 3400 ×*g* for 30 min. The supernatant was discarded leaving a pellet, further environmental sample was added to the centrifuge tube and the process repeated until 5 L sample had been processed. The pellet was subsequently resuspended in 50 mL supernatant.

### 2.5 Column colonisation (CC)

A method previously described by Mauffret et al. (2009) for enrichment of biofilms onto glass beads was followed with some modifications. Sterile 0.4 mm glass beads were packed into a 50 mL syringe sealed with 5 μm cloth and cotton wool at the bottom and a rubber bung at the top, through which the environmental sample was fed via plastic tubing using a peristaltic pump. The environmental sample was passed through the column at a rate of 15 mL min^−1^ and recirculated to the feed tank over a period of 10 days. The period of colonisation was reduced from 72 days based on a 30 day study indicating that a stable concentration of cells was established on the beads after a 10 day period (data not shown). At the end of the colonisation period, the glass beads were sonicated in 50 mL of the respective environmental sample, encouraging the resuspension of biomass, yielding a concentrated test inoculum (Mauffret et al., 2009).

### 2.6 High-throughput biodegradation screening test (HT-BST)

250 μL 4-nitrophenol (10 mg C L^−1^ in OECD mineral media (OECD, 1992)) was added to each well of a 96-well plate as the sole carbon source. Concentrated inocula (termed 10^0^) were serially diluted to give five dilutions (termed 10^−1^ to 10^−5^). Cell concentrations ranged from 10^3^ to 10^8^ cells mL^−1^, where 10^0^ refers to 10^8^ cells mL^−1^. 96-well plates were inoculated with 50 μL of the desired cell concentration before being closed and sealed with Parafilm (Pechiney Plastic Packaging Company, Chicago, IL). Sealed plates were placed on top of water saturated filter papers lining a plastic box, which was subsequently sealed with cling film to create a moisture chamber, reducing the risk of evaporation. The box was wrapped in tin foil to prevent photo interference and photodegradation and incubated at 20 °C for 60 days (Goodhead et al., 2013; Martin et al., 2017a).

### 2.7 Biodegradation determination and interpretation

4-nitrophenol transforms from a yellow to a colourless solution on degradation, which was determined against a calibration curve of 4-NP standard concentrations (1–35 mg L^−1^) at an optimum wavelength determined as 400 nm. The calibration curve showed linearity below the 3 mg C L^−1^ required to detect a 70% reduction in chemical concentration in the prescribed study. After 60 days, biodegradation was determined by colorimetric detection of residual parent compound in each microtitre well using a Multiskan® Spectrum spectrophotometer (Thermo Scientific, Waltham, Massachusetts, USA). A positive response was defined as 30% or less of the initial chemical remaining, which equates to the 70% DOC removal level demarcated in OECD 301 guidelines for the testing of chemical ready biodegradability (OECD, 1992). Probability of degradation was then calculated based on the frequency of positive wells for each inocula concentration for each environmental compartment and cell concentration method. 4-nitrophenol is classified as non-persistent and inherently or variably biodegradable, according to its ECHA Brief Profile Classification (OECD, 2013), the reliability of the HT-BST to reflect this classification was assessed, in addition to the outcome of the test.

### 2.8 Bacterial community analysis

An approach using both denaturing gradient gel electrophoresis (DGGE) and 454 pyrosequencing was adopted to assess community similarity and diversity. DGGE has traditionally been used in microbial molecular ecology to analyse community composition (Goodhead et al., 2013). Amplified DNA fragments are separated by electrophoresis based on differences in sample sequence, resulting in a banding pattern indicative of the predominant bacteria in a community whereby each band represents and operational taxonomic unit (OTU). Recent advancements in sequencing throughput, and reductions in sequencing costs, have led to conventional molecular fingerprinting techniques being progressively replaced by high throughput sequencing methods. However, when used as a complementary tool to more in-depth sequencing efforts such as 454 pyrosequencing, DGGE adds experimental value by providing rapid, relatively cheap, analysis of variations in community composition (Cleary et al., 2012).

#### 2.8.1 DNA extraction

Total DNA was extracted from triplicate 250 μL subsamples of each sample before and after cell concentration using FastDNA SPIN kit for soil (BP Biomedicals, Solon, Ohio, USA) by a combination of chemical and physical lysis, as detailed by Baptista et al. (2008).

#### 2.8.2 Denaturing gradient gel electrophoresis

16S rRNA gene fragments were amplified by PCR using primers and conditions specified by Muyzer et al. (1993), targeting conserved regions of the gene (Primer 2: 5′-ATTACCGCGGCTGCTGG-3′; Primer 3: 5′-*CGCCCGCCGCGCGCGGCGGGCGGGGCGGGGGCACGGGGGG*CCTACGGGAGGCAGCAG-3′). Primer 3 contains an additional 40-nucleotide GC-rich sequence (GC clamp) (Muyzer et al., 1993). PCR reactions were carried out in 200 μL PCR tubes, containing 1 μL template DNA, 10 pmol (in 1 μL) of each primer and 47 μL of MegaMix-Blue PCR-ready mix (containing *Taq* polymerase in 1.1 × reaction buffer (2.75 mM MgCl_2_) with 220 μM dNTPs, loading dye and stabiliser: Microzone, HaywardsHeath, UK). PCR was performed using a Thermo-cycler (Thermo Fisher Scientific, Waltham, MA, USA), with the following amplification conditions: an initial denaturation phase at 95 °C at 3 min prior to a one minute denaturation step at 95 °C, followed by one minute cycle at 65°, reducing by 1 °C every second cycle until 53 °C, at which stage 15 additional cycles were performed and a final extension step of 72 °C for 10 min. DGGE was performed as described previously (Green et al., 2009; Goodhead et al., 2013) using the D-code system (Bio-Rad, Hemel Hempstead, UK). 10% polyacrylamide gels (0.75-mm thick, 16 × 16 cm) were run in 1 × TAE buffer (40 mM Tris-Acetate, 1 mM EDTA, pH 8.3). A gradient of 30–60% denaturant was used (where 100% denaturant contains 7 M urea plus 40% v/v formamide in 1 × TAE). Gels were run at 60 °C for 900 V hours (approximately 4.5 h at a constant voltage of 200 V). Gels were subsequently stained for 30 min using SYBR Gold (diluted to 1:10,000 in 1 × TAE: Sigma Aldrich, St. Louis, MO, USA) and imaged using a UV transilluminator.

#### 2.8.3 Pyrosequencing

16S rRNA gene fragments were amplified by PCR using MID (multiplex identifier) primers, allowing for identification of samples downstream. The F515 (5′- GTGNCAGCMGCCGCGGTAA-3′) and R926 (5′-CCGYCAATTYMTTTRAGTTT-3′) were attached to an adaptor sequence (CGTATCGCCTCCCTCGCGCCATCAG and CTATGCGCCTTGCCAGCCCGCTCAG respectively) and a unique 8-base barcode sequence to allow sample differentiation in downstream processes. PCR reactions were carried out in 200 μL PCR tubes, containing 1 μL template DNA, 1 μL of each primer (with respective barcode attached), 5 μL Fast Start High Fidelity buffer, 0.5 μL Fast Start High Fidelity enzyme blend, 0.5 μL PCR nucleotide mix (all Roche Applied Science, Penzberg, Germany), and made up to a 50 μL reaction volume with sterile Milli-Q water. PCR was performed using a Thermo-cycler (Thermo Fisher Scientific, Waltham, MA, USA), with the following amplification conditions: an initial denaturation phase at 95 °C at 4 min prior to 25 cycles of 1 min at 95 °C, 45 s at 55°, 1 min at 72 °C and a final extension step of 72 °C for 7 min. PCR products were purified using the MinElute PCR purification kit (Qiagen, Limburg, Netherlands) as per the manufacturer's instructions, with elution into a final volume of 20 μL. DNA quantitation was performed using the Qubit 2.0 fluorometer (Life Technologies, Paisley, UK) as per the manufacturer's instructions. The presence of DNA was analysed on a 1% agarose gel as previously described. Samples were prepared in an equimolar amplicon pool (based on the measured DNA concentrations), which was then sent to the Centre for Genome Research at Liverpool University for processing on a 454 Genome Sequencer (GS) FLX platform with titanium chemistry.

Analysis was conducted using the open source software package, Quantitative Insights Into Microbial Ecology (QIIME, Version 1.7) (Caporaso et al., 2010) Noise was removed from sequencing data using AmpliconNoise (Quince et al., 2011). Similar sequences were grouped and assigned to operational taxonomic units (OTUs) using Uclust (Edgar, 2010), based on 97% similarity. Representative sequences were aligned against the SILVA reference database, version 119 (Quast et al., 2013). Prior to community similarity analysis, OTU tables were filtered by abundance to leave only OTUs present at an abundance of 0.01% or greater across the relevant samples. Statistical analyses were performed using PRIMER (PRIMER 7, version 7.0.11, PRIMER-E Ltd., Plymouth, UK) (Clarke et al., 2014).

### 2.9 Method ranking

The different cell concentration methods were ranked based on scientific and practical criteria. The scientific criteria were: final cell concentration achieved, similarity of the concentrated communities to those in the original sample, the diversity of the concentrated community and the reliable assessment of biodegradation potential of 4-nitrophenol. The practical criteria were: equipment cost, operational and maintenance costs, potential sample throughput (function of volume and time) and the level of skill required to implement the procedure. The highest ranked method in each category was awarded a score of 1, and the lowest-ranking method was given a score of 4. Average ranks were calculated based on the cumulative rank scores for scientific and practical criteria separately and for their combined scores overall. No preferential weighting was given to any of the criteria.

## 3 Results and discussion

### 3.1 Cell concentration

**Fig. 1:**
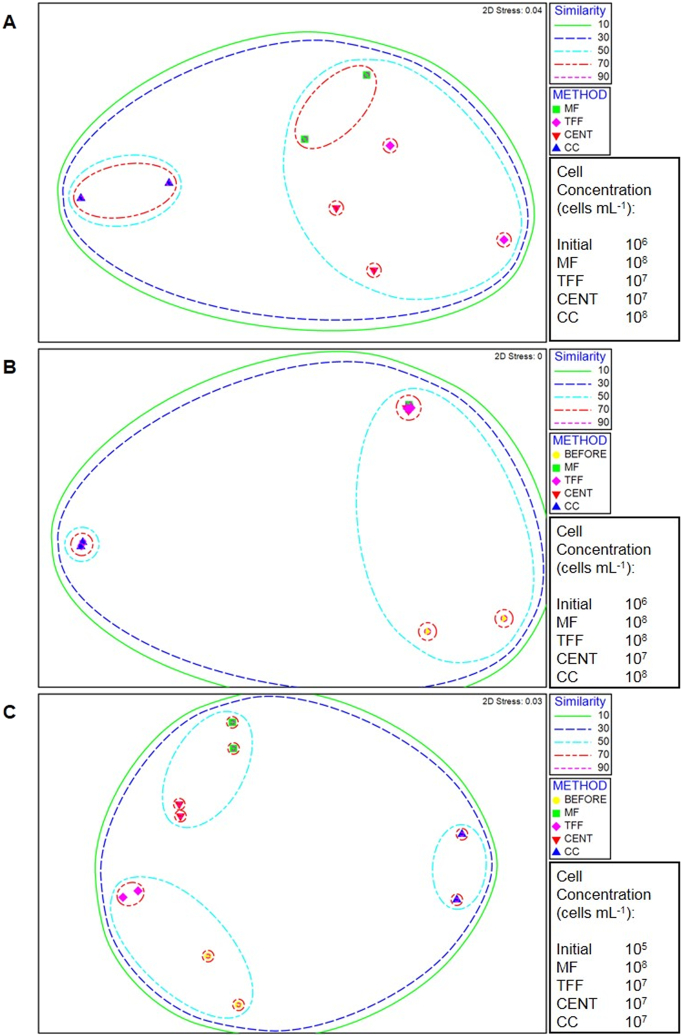
Non-metric multidimensional scaling plots overlaid with cluster analysis threshold similarities for 454 sequencing data, detailing the similarity between microbial communities of inocula from (A) river; (B) estuarine; and (C) marine samples from the original (BEFORE) sample and from the highest concentration of inocula achieved by membrane filtration (MF), tangential flow filtration (TFF), centrifugation (CENT) or column colonisation (CC) from the same sample. No before sample was used for the river compartment, due to a PCR fail in the original sample. Initial cell concentrations and maximum cell concentrations achieved for the different methods are included alongside the NMDS plots for each environmental compartment.

Cell concentration was successfully increased for all methods, by as little as 10-fold (centrifugation) to as much as 1000-fold (membrane filtration), in seawater, based on total cell counts via epifluorescence microscopy (Fig. 1). Membrane filtration consistently ranked highest with respect to cell concentration, regardless of the aqueous sample used. Membrane filtration, tangential flow filtration and column colonisation produced maximum inocula concentrations of 10^8^ cells mL^−1^; centrifugation produced a maximum inocula concentration of 10^7^ cells mL^−1^. Column colonisation also gave high cell concentrations in contrast to centrifugation, which concentrated fewer cells than any other method probably because many bacterioplankton are present as small dispersed unicells possessing a low specific gravity. According to Stoke's law such samples would require high centrifugal forces and long centrifugal times to overcome buoyancy and viscosity forces. In contrast to the method here, ultracentrifugation may be successful in concentrating such cells, but is more costly than conventional centrifugation and may negatively impact cell viability (Børsheim et al., 1990). Moreover, in aquatic microbiology, centrifugation is seldom used and often criticized for its impracticality and inability to capture small cells (Šimek and Straškrabová, 1992; Lemke et al., 1997).

### 3.2 Bacterial community analysis

The most important scientific criterion is the ability to increase bacterial cell concentration without adversely changing the community structure of the predominant taxa present in the original sample. Concentrated inocula should therefore contain the same taxa in approximately the same proportions as in the original samples, but also include the presence of additional rarer taxa excluded from smaller dilute sampling strategies. Such bacterial communities were expected to retain a large number of taxa present in the original samples, and have greater taxa richness than the original community.

The similarity of bacterial communities within replicate samples derived from the same cell concentration method were significantly greater than those similarities between different cell concentration methods for both 454 and DGGE analysis (ANOSIM, *P* < 0.001), with the majority of replicates (14 out of 14, and 13 out of 14 for 454 and DGGE respectively) showing >60% similarity in bacterial community composition (Fig. 1; Fig. SI S2). It is perhaps unsurprising that those methods based on size exclusion (i.e. membrane filtration and tangential flow filtration) gave bacterial communities that were most similar to the original communities. Column colonisation resulted in the lowest bacterial community similarity with the original sample (Fig. 1). A proxy sample used in place of the original failed river sample, sampled from the same location but at a different time, was originally included in the similarity analysis (Fig. SI S3) but removed as the temporal variation appeared to skew the community similarity analysis. Community similarity from 454 sequencing was derived from presence/absence resemblance matrices, considering only OTUs present at an abundance of 0.01% or greater. Community similarity plots without the abundance cut-off are presented in SI (Fig. SI S4).

As a result of the increasing cell concentration evident across the river, estuarine and marine inocula (Fig. 1), the diversity of the samples might also be expected to increase. In DGGE gels, a band represents a taxon and the number of bands therefore represents the OTU richness for the most abundant taxa in a given sample. Generally, membrane filtration, tangential flow filtration and centrifugation produced bacterial communities with OTU richness values similar to, and often greater than, the original native sample. Following data aggregation, only membrane filtration and tangential flow filtration had mean values (20.6 and 24.8 respectively) greater than the original sample (20.5) whilst centrifugation averaged 19.7. All four samples were statistically indistinguishable from each other but significantly different from column colonisation, which had an OTU richness of only 10.6 (ANOVA, *P* < 0.001). Such differences were less noticeable when using 454 pyrosequencing data (see below). Column colonisation often produced communities where some of the resulting dominant taxa were not those that dominated in the original sample; in some cases column colonisation enriched rare taxa that appeared absent in the original sample (Fig. SI S2). This is consistent with a method selectively enriching only a proportion of the original community, which most likely consists of only those taxa capable of forming biofilms. Such communities are misrepresentative of the majority of the natural aqueous environment, which is an undesirable trait when investigating the biodegradation potential of predominantly pelagic communities. For marine samples, tangential flow filtration was the only method that increased OTU richness beyond that exhibited in the original sample; the other methods reported reductions in OTU richness. For estuarine samples, all methods increased the OTU richness compared to the original sample. For river samples, centrifugation and tangential flow filtration gave the highest OTU richness values followed by membrane filtration (30, 28.7, and 21.7, respectively), and glass bead column colonisation had a significantly 3-fold lower OTU richness (7) than membrane filtration (*t*-test, *P* < 0.01). PCR failure prevented a comparison of the concentration methods with the original sample from the river in this case. When a sample from the same location, but taken at a different time to the others, was used as a proxy for the original sample, centrifugation, tangential flow filtration and membrane filtration gave higher taxa richness values than the proxy.

Following data aggregation, 454 sequencing produced mean Chao1 richness estimates for membrane filtration, tangential flow filtration and column colonisation (1000, 997 and 1000 taxa respectively) similar to the original samples (998 taxa), whilst centrifugation averaged 856 taxa (Table SI S1). Theoretically, diversity should increase as the cell concentration increases. This anticipated increase in diversity, however, was not reflected in high throughput sequencing of the concentrated samples. This observation is believed to be due to the relatively low sequencing depth (average 6100 reads per sample) used in the present study, which was probably insufficient to explore the true diversity of the environments sampled. Whilst this depth was sufficient to make assessments of community similarity, a more rigorous sequencing effort would be required in future work to elucidate the impact of cell concentration on alpha diversity.

### 3.3 Impact on biodegradation outcome

**Fig. 2:**
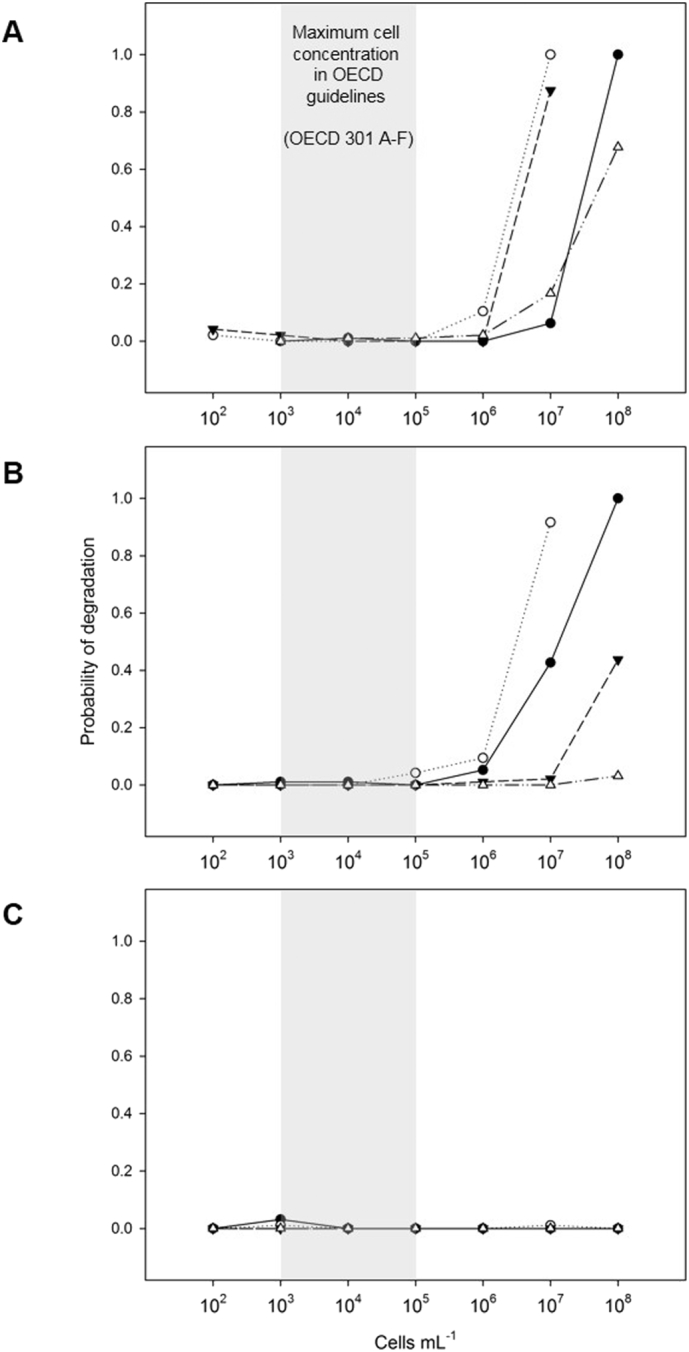
The biodegradation potential of 4-nitrophenol in 96-well plate high throughput assays for environmental inocula (*a*. River; *b*. Estuarine; *c*. Marine) at different inocula concentrations achieved by dilution or by concentration using different techniques: Membrane Filtration ●; Centrifugation ○; Tangential Flow Filtration ▼; Column Colonisation △.

The test compound 4-nitrophenol, is classified as non-persistent and inherently or variably biodegradable, according to ECHA Brief Profile Classifications (OECD, 2013). This assessment is supported by a body of research, showing variable degradation linked to inoculum concentration (Thouand et al., 1995; Martin et al., 2017a; Martin et al., 2017b). The lowest cell concentration resulting in a high probability of degradation (100%) was observed in inocula derived from concentrating cells by centrifugation (10^7^ cells mL^−1^ with river and estuarine inocula), followed by tangential flow filtration (90% probability of degradation at 10^7^ cells mL^−1^ with river inocula) and membrane filtration (100% probability of degradation at 10^8^ cells mL^−1^ with river and estuarine inocula) (Fig. 2; Table SI S1). Column colonisation showed a relatively high probability of degradation at higher inoculum concentration in river water HT-BSTs, but very low probability of degradation for concentrated estuarine samples (Fig. 2). The poor probability of degradation may be related to the shift in community composition observed due to the preparation bias, selectively enhancing certain bacteria and potentially excluding competent degraders of the test compound present in the original sample.

The likelihood of reliably making the correct classification of biodegradation potential for the inherently or variably biodegradable 4-nitrophenol was related to the statistical power of the test. If we consider the power of the test to be 1 minus the type II error, and the type II error to be the likelihood of accepting a test fail when we know the chemical is classified as inherently biodegradable, then the power of the test is greatest at the highest inoculum concentration (10^8^ cells mL^−1^) for inocula derived from estuarine and river environments. The test has the greatest power with inocula concentrated by centrifugation and membrane filtration (1 for inocula derived from river and estuarine sources), then tangential flow filtration (1 for inocula derived from river sources). Tests incorporating inocula concentrated by column colonisation typically had a low statistical power, not exceeding 0.25 (Fig. SI S5). This suggests that the method of concentration, and not just the total number of cells within a test, plays an important role in making reliable characterisations of biodegradation potential.

The cell concentration range tested in this study covers the concentration range typically used within OECD biodegradability screening tests (10^3^–10^5^ cells mL^−1^, e.g. OECD 301, 306), extending to concentrations which would typically be found in the natural environment (10^5^–10^7^ cells mL^−1^) and to environmentally relevant concentrations which may be encountered in wastewater treatment plants (10^8^–10^9^ cells mL^−1^). The probability of degradation increases in the HT-BST as the cell concentration increases, with more environmentally relevant inoculum concentrations resulting in a more reliable assessment of biodegradation. This increase in confidence when assessing chemical biodegradability using higher inoculum concentrations reflects observations reported in larger scale tests (Martin et al., 2017b).

The HT-BSTs showed no degradation of the test compound, 4-NP, at any concentration of marine inocula (Fig. 2). It is suggested that this is not necessarily an indication of the biodegradation potential of 4-NP; it may be one of test duration. Extended lag phases have been reported for 4-NP previously (Nyholm et al., 1992; ECETOC, 2017), linked to the competent degraders required for biodegradation to occur, being present at very low initial population densities. Additional confounding factors may also have an impact to the marine HT-BST particularly, for example the 250 μL sterile mineral media may be inducing an osmotic shock in the marine microorganisms.

### 3.4 Practical criteria

**Fig. 3:**
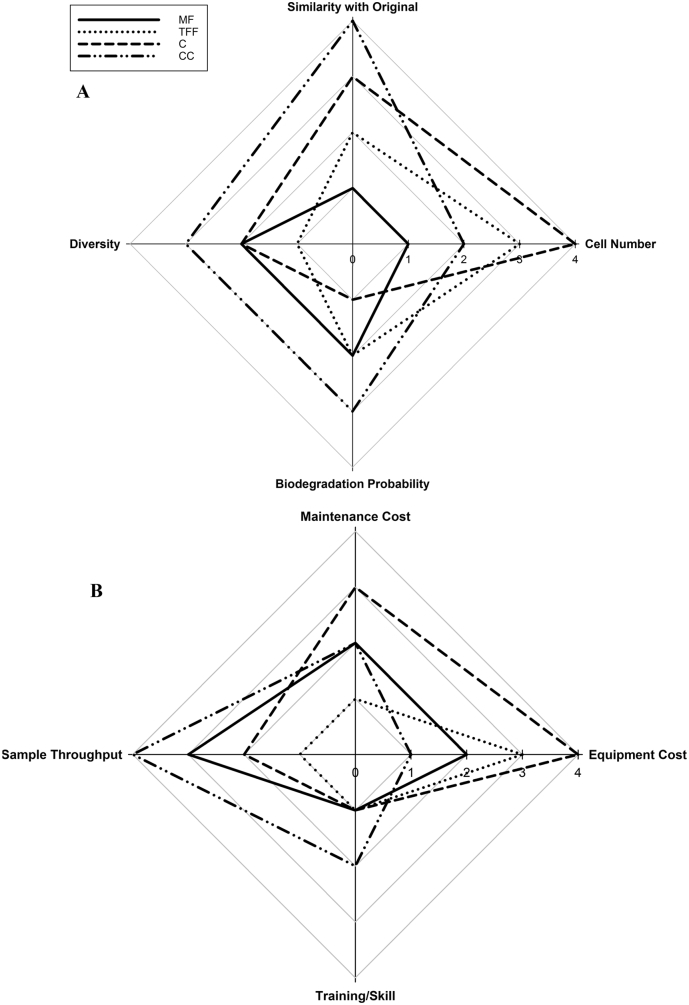
Rankings for concentration methods considering performance in river, estuarine and marine samples against (A) scientific criteria and (B) practical criteria. Proximity to the centre of the plot indicates the optimum method. Rankings for individual compartments are shown in Table SI S3.

A summary of the practical considerations are given in Table 1 and Fig. 3. Briefly: the initial equipment outlay is greatest for centrifugation and tangential flow filtration; column colonisation requires the greatest level of skill and training to successfully concentrate bacterial cells; and sample throughput is greatest for tangential flow filtration. A complementary tangential flow filtration method has since been developed allowing the recovery of bacterial cells from the filter surface without the backwashing step, which better maintains the integrity of the filters and improves their longevity (data not shown).

**Table 1:** Summary of practical criteria to be considered during method selection. All costs are indicative, based on UK estimates.

Practical consideration	MF	TFF	C	CC
Availability of equipment within environmental laboratory	Standard lab equipment	TFF manifold not standard equipment	Standard lab equipment although existing centrifuge capacity may not be suitable	Standard lab equipment. Glass beads may need purchasing
Cost (assuming best and worst case scenario)	$1300–$6500	$6200–$25,800	$5200–$25,800	$130–6500
Operational and maintenance requirements	Vacuum pump; Filters	Peristaltic pump; *Re*-useable filters; Cleaning/Sanitising solutions; Periodic tubing replacement	Yearly service contracts ($1300 - $2600 p.a.); Replacement components; Electricity cost	Peristaltic pump; Syringes; Glass beads; Plastic tubing
Sample throughput (estimated in L h^−1^)	1.7	2.5–60	5	0.007–0.01

MF = membrane filtration; TFF = tangential flow filtration; C = centrifugation; CC = column (glass bead) colonisation.

### 3.5 Overall method ranking

Concentration methodologies were ranked according to their performance for all of the scientific and practical criteria described above in the context of providing inocula for biodegradation tests. A score of 1 was assigned to the best performing procedure(s) and protocols were ranked based on their average rank, with a lower overall average being the most desirable outcome (Fig. 3).

Based on the scientific criteria, membrane filtration would be the best concentration method to prepare inocula for biodegradation tests, scoring the highest average rank. The highest average rank with respect to practical criteria was attributed to tangential flow filtration. Overall, tangential flow filtration was designated as the highest ranked method, closely followed by membrane filtration. Column colonisation was the lowest ranked method overall, performing particularly poorly with respect to the scientific criteria (Fig. 3). It is worth noting that, whilst no preferential weightings were given to any of the criteria selected, if the main criterion is considered to be the community similarity of the concentrated sample to the original unprocessed sample, and the sample throughput, those methods ranked highest would still be the best performing, highest ranking systems.

Based on the chosen selection criteria, it is recommended that there is no single concentration method fit for all purposes but rather the appropriate method should be selected on a case-by-case basis. The low ranking of column colonisation, particularly with respect to community similarity and its logistical infeasibility at laboratory scale, preclude this method from selection for planktonic applications. The remaining methods, however, have their own role within cell concentration for biodegradation test inocula. Membrane filtration would be the best method when biomass concentrations are not excessively high and relatively small volumes (up to 5 L) are being processed. The increased throughput capability of tangential flow filtration render it the most suitable method when processing large sample volumes, and centrifugation would be the most appropriate method for samples with high initial biomass concentrations, such as activated sludge.

## 4 Conclusions

• Low inoculum concentrations used in ready biodegradability tests may lead to the exclusion of relatively rare competent degraders;
• Methods are available to concentrate aqueous environmental samples whilst preserving similarity to the original microbial community;
• Increased inoculum concentrations result in increased probabilities of degradation for readily or inherently biodegradable compounds and provide (i) a more effective approach to differentiating between persistent and non-persistent chemicals and (ii) fewer false negative assignments of a chemicals degradability;
• The cell concentration method employed should be selected on a case by case basis, considering initial concentration and sample throughput;
• Column colonisation produces communities with poor similarity to the original community, possibly due to selective bias of organisms capable of forming biofilms;
• More rigorous investigative efforts are required to interrogate diversity in environmental samples;
• High throughput biodegradation screening tests are a useful tool for investigating environmental factors affecting biodegradation outcome.

## Appendix A Supplementary data

Supplementary material Image 1
