# Supplementary material for: Improving the ecological relevance of aquatic bacterial communities in biodegradability screening assessments
Source: Sci Total Environ. 2018 Jun 15;627:1552–9. doi: 10.1016/j.scitotenv.2018.01.264 (PMC5892456; doi:10.1016/j.scitotenv.2018.01.264)
Supplement: Supplementary file 1 — Supplementary material [file mmc1.docx]

Improving the ecological relevance of aquatic bacterial communities in biodegradability screening assessments

Timothy J. Martin ^a^*, Andrew K. Goodhead ^a, 1^, Jason R. Snape ^a, b, c^, Russell J, Davenport ^a^

^a^ School of Engineering, Cassie Building, Newcastle University, Newcastle upon Tyne, NE1 7RU, United Kingdom.

^b^ AstraZeneca Global Environment, Mereside, Alderley Park, Macclesfield, Cheshire, SK10 4TG, United Kingdom.

^c^ School of Life Sciences, Gibbet Hill Campus, The University of Warwick, Coventry, CV4 7AL.

*Corresponding author: timothy.martin@ncl.ac.uk; Telephone +44(0)1912087091; Fax +44(0)1912086502

^1^ Present address: Andrew K. Goodhead is presently affiliated with Promega Corporation, Southampton, UK.

**SUPPORTING INFORMATION**


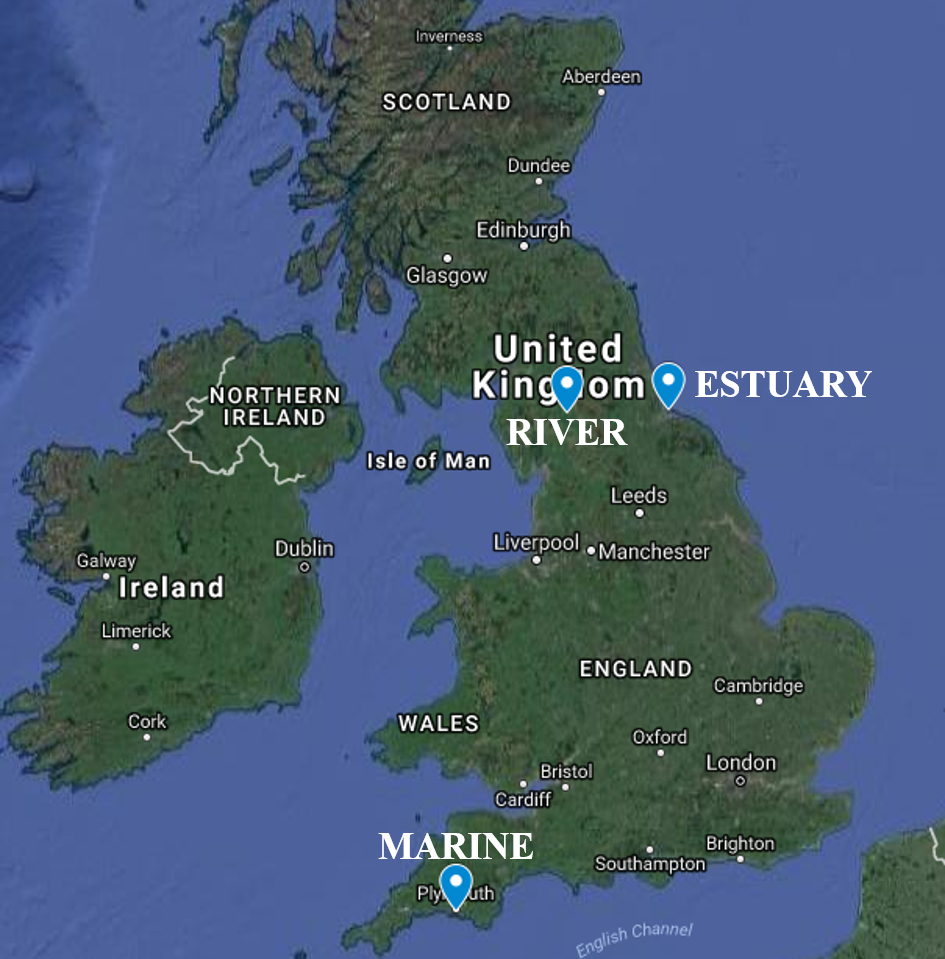


**Figure SI S1.** Sampling locations at Temple Sowerby (river), Teesmouth (estuary) and Plymouth (marine).

***A***

***B***


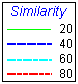

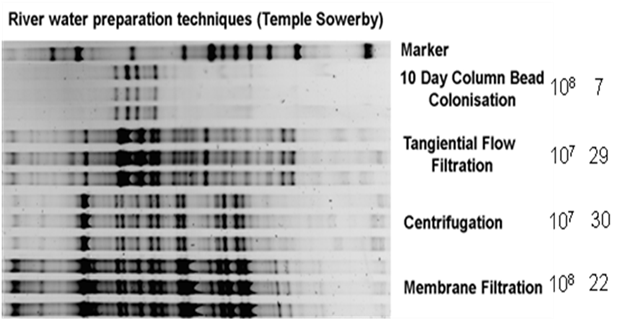

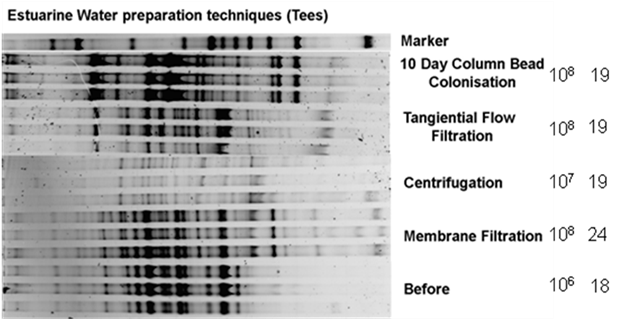

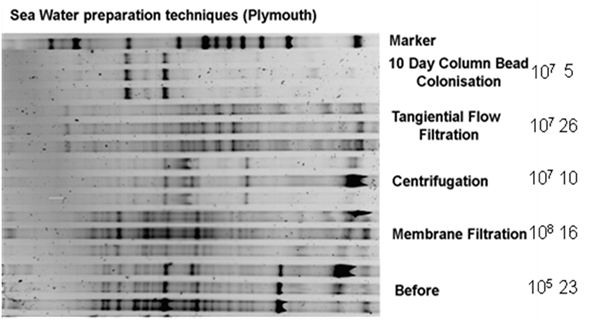


*i.*

*iii.*

*ii.*

**Figure SI S2**. (**A**) Non-metric multidimensional scaling plots overlaid with cluster analysis threshold similarities, detailing the similarity of microbial communities from an original sample with those prepared by concentrating cells using different methods, based on DGGE community analysis (**B**). *i.* River; ii*.* Estuarine; *iii.* Marine. Where, Before ; Membrane Filtration ; Centrifugation ; Tangential Flow Filtration ; Column (Glass Bead) Colonisation .

**
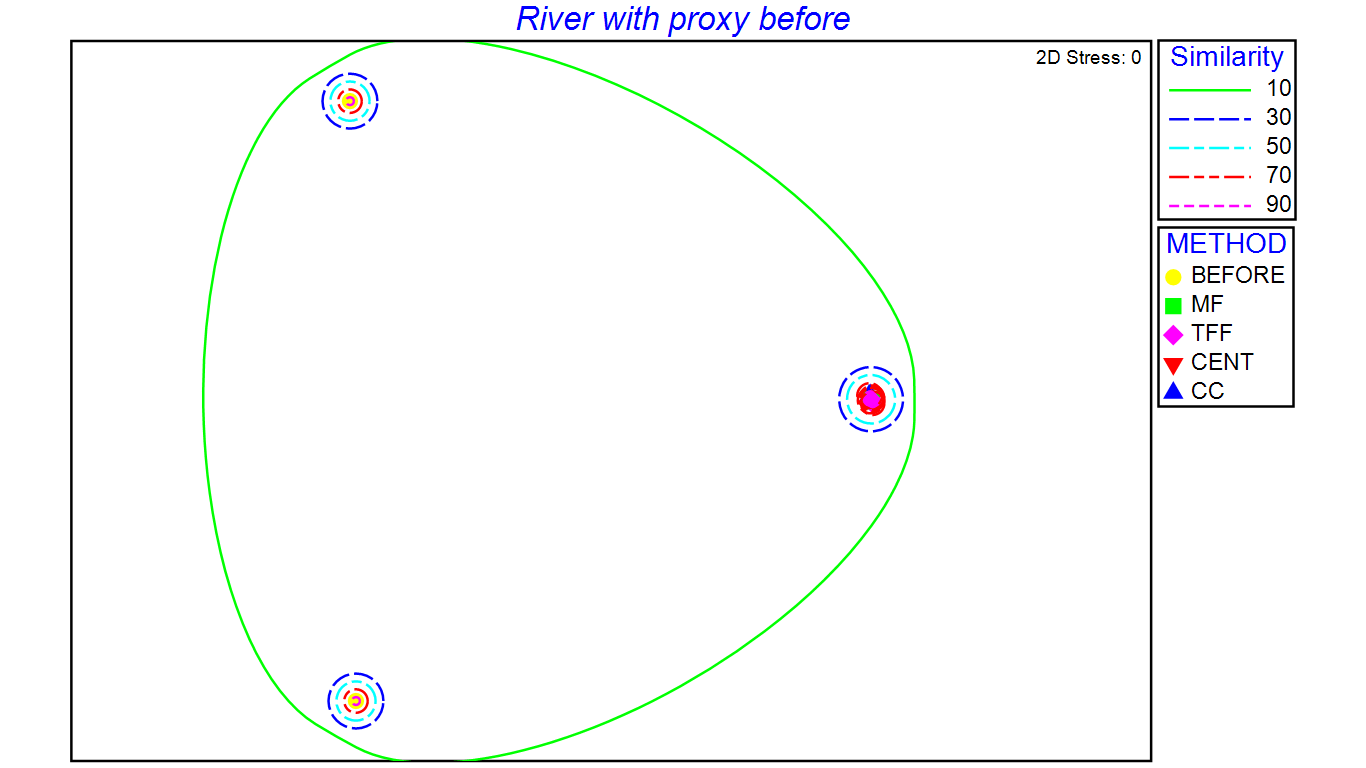
**

**Figure SI S3.** Non-metric multidimensional scaling plots overlaid with cluster analysis threshold similarities, detailing the similarity between microbial communities based on 454 pyrosequencing community analysis from an original sample and from those concentrated using different methods. A proxy BEFORE sample was taken from the same sampling location was used due to a PCR failure in the original sample, however the temporal variation resulted in a considerable dissimilarity between the proxy sample and the concentrated sample, therefore the proxy sample was removed for the purposes of similarity analysis. The proxy sample is included here to highlight its impact on community similarity analysis. KEY: Before = original sample; MF = Membrane Filtration; TFF = Tangential Flow Filtration; CENT = Centrifugation; CC = Column (Glass Bead) Colonisation.


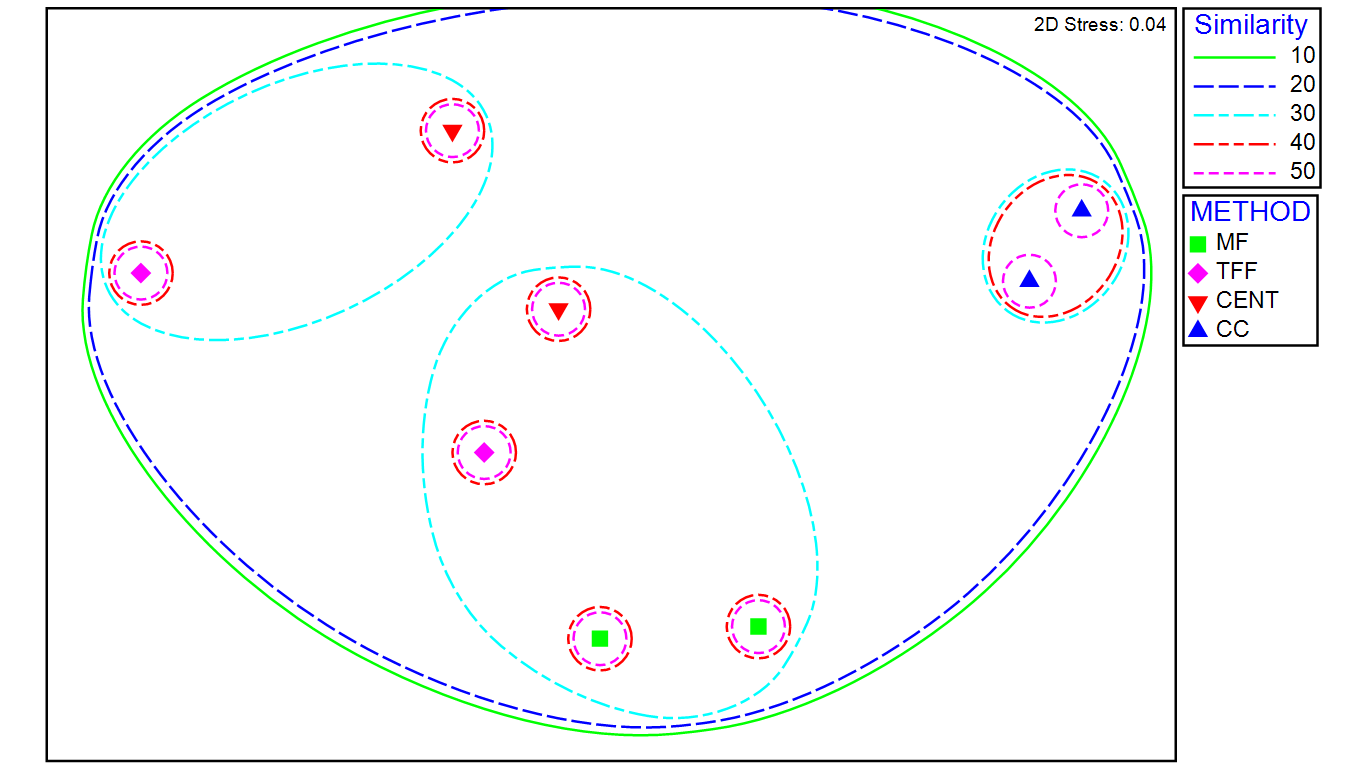

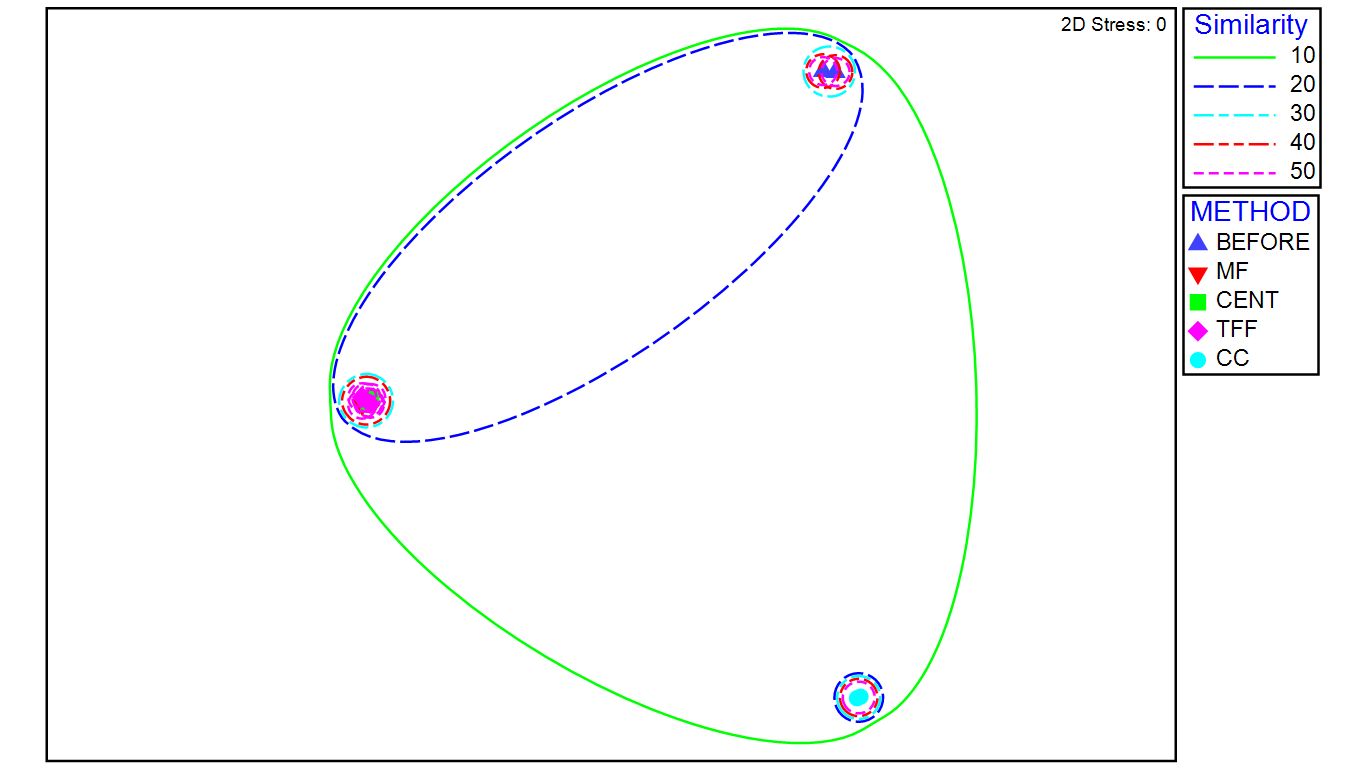

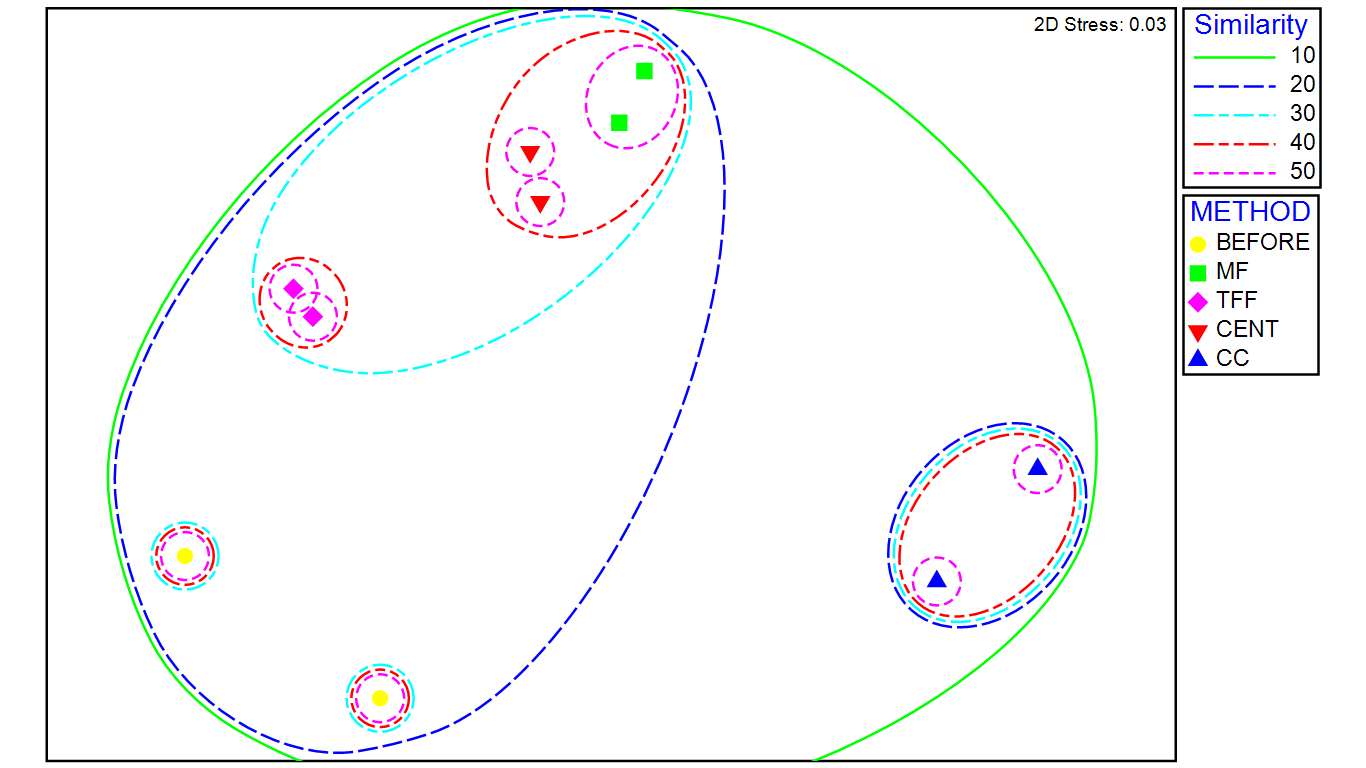


*i.*

*ii.*

*iii.*

**Figure SI S4.** Non-metric multidimensional scaling (nMDS) plots overlaid with cluster analysis threshold similarities, for microbial communities analysed using 16S amplicon NGS from (*i.*) river, (*ii.*) estuarine and (*iii.*) marine samples without taxa abundance filtering. General trends observed in Figure 1 are still evident but levels of similarity are proportionally reduced across the samples due to the inclusion of rare organisms. These differences are amplified by the presence/absence nature of the resemblance matrix used to create the nMDS plots, increasing the influence low count OTUs have on similarity. KEY: Before = original sample; MF = Membrane Filtration; TFF = Tangential Flow Filtration; CENT = Centrifugation; CC = Column (Glass Bead) Colonisation.


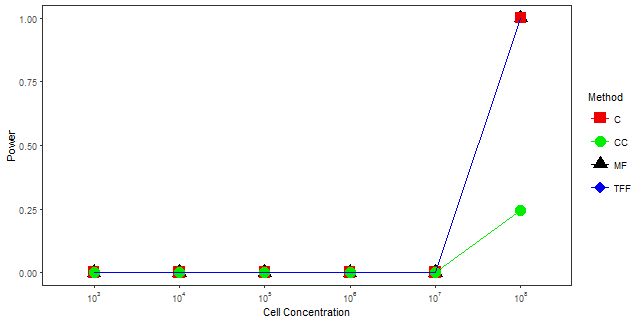

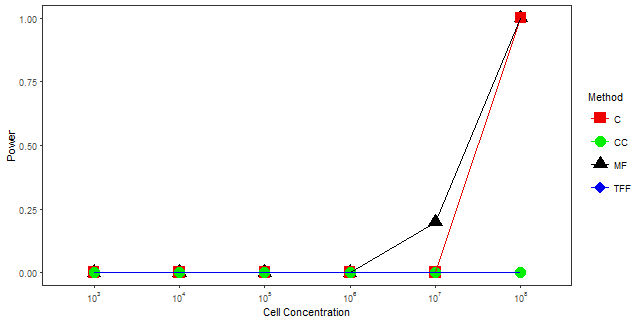

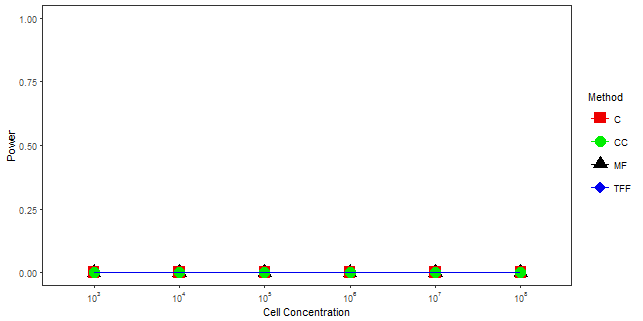


*River*

*Estuary*

*Marine*

**Figure SI S5.** Statistical power against cell concentration (cells mL^-1^), for the biodegradation assessments incorporating inocula from the four tested concentration methods, sourced from river, estuarine and marine environments. The likelihood of observing a false negative, where the inherently biodegradable 4-nitrophenol fails to degrade in a biodegradation assessment, was related to the statistical power of the test. The power of the test is calculated as 1 minus the type II error, and type II error is considered the likelihood of accepting a test fail when degradation might be expected, given the inherently biodegradable classification of the compound.

**Table SI S1.** Summary of cell concentration and OTU richness measures, determined using epifluorescence microscopy and DGGE and 454 sequencing respectively, combined with downstream processing. *indicates a proxy sample was used, due to a PCR fail in the original sample, this proxy sample was taken from the same sample location but at a different time to the original sample

|  | **River** | | | **Estuarine** | | | **Marine** | | |
| --- | --- | --- | --- | --- | --- | --- | --- | --- | --- |
|  | Cells/mL | Richness | | Cells/mL | Richness | | Cells/mL | Richness | |
|  |  | DGGE | 454 Chao1 estimate | DGGE | DGGE | 454 Chao1 estimate |  | DGGE | 454 Chao1 estimate |
| Membrane filtration | 10^8^ | 22 *(±1.2)* | 1980 | 10^8^ | 24 *(±1.0)* | 706 | 10^8^ | 16 *(±0.0)* | 313 |
| TFF | 10^7^ | 29 *(±0.6)* | 1195 | 10^8^ | 19 *(±1.2)* | 1035 | 10^7^ | 26 *(±1.5)* | 760 |
| Centrifugation | 10^7^ | 30 *(±2.0)* | 1124 | 10^7^ | 19 *(±1.2)* | 905 | 10^7^ | 10 *(±0.6)* | 541 |
| Column Colonization | 10^8^ | 7 *(±0.0)* | 1674 | 10^8^ | 19 *(±0.6)* | 910 | 10^7^ | 5 *(±0.6)* | 416 |
| Original | 10^6^ |  | 557***** | 10^6^ | 18 *(±4.5)* | 1203 | 10^5^ | 23 *(±0.6)* | 1015 |

**Table SI S2.** Summary of method ranking based on DGGE and 454 sequencing community analysis and biodegradation potential analysis.

| **Sample** | Bacterial community  similarity | | Cell concentration | Band richness | | Greatest probability at lowest [cell] |
| --- | --- | --- | --- | --- | --- | --- |
|  | DGGE | 454 |  | DGGE | 454 |  |
| River | PCR Fail | PCR Fail | MF=CC>TFF=C | C>TFF>*MF*>CC | MF>CC>TFF>C | C>TFF>MF>CC |
| Estuarine | MF>*TFF*>C>*CC* | MF=TFF=C>CC | MF=TFF=CC>C | MF>TFF=C=CC | TFF>CC>C>MF | C>MF>TFF>CC |
| Marine | MF>*TFF*>C>*CC* | TFF>C>MF>CC | MF>TFF=C=CC | TFF>*MF*>C>*CC* | TFF>C>CC>MF | C=TFF=MF=CC |
| **C=centrifugation; MF=membrane filtration; TFF=tangential flow filtration; CC=column colonization. Differences in font colour show treatments which gave statistically significant differences** | | | | | | |

**Table SI S3.** Individual environmental compartment method rankings for scientific and practical criteria. Average rankings across the compartments were used to rank the methods (Figure 3).

| **Scientific Criteria** | | | | |
| --- | --- | --- | --- | --- |
| ***River*** | **Cell number** | **Similarity with original** | **Diversity** | **Probability of Degradation** |
| C | 2.00 | 3.00 | 1.00 | 1.00 |
| MF | 1.00 | 1.00 | 3.00 | 3.00 |
| TFF | 2.00 | 2.00 | 2.00 | 2.00 |
| CC | 1.00 | 4.00 | 4.00 | 4.00 |
| ***Estuarine*** | |  |  |  |
| C | 2.00 | 3.00 | 2.00 | 1.00 |
| MF | 1.00 | 1.00 | 1.00 | 2.00 |
| TFF | 1.00 | 2.00 | 2.00 | 3.00 |
| CC | 1.00 | 4.00 | 2.00 | 4.00 |
| ***Marine*** |  |  |  |  |
| C | 2.00 | 3.00 | 3.00 | 1.00 |
| MF | 1.00 | 1.00 | 2.00 | 1.00 |
| TFF | 2.00 | 2.00 | 1.00 | 1.00 |
| CC | 2.00 | 4.00 | 4.00 | 1.00 |
|  |  |  |  |  |
| **Practical Criteria** | | | | |
| **Method** | **Equipment cost** | **Maintenance costs** | **Sample throughput** | **Training/Skill** |
| C | 4.00 | 3.00 | 2.00 | 1.00 |
| MF | 2.00 | 2.00 | 3.00 | 1.00 |
| TFF | 3.00 | 1.00 | 1.00 | 1.00 |
| CC | 1.00 | 2.00 | 4.00 | 2.00 |
